# Successful Conversion Surgery after Zolbetuximab-Based Chemotherapy for Claudin 18.2-Positive Gastric Cancer with Peritoneal Dissemination and Cervical Lymph Node Metastasis: A Case Report

**DOI:** 10.70352/scrj.cr.25-0536

**Published:** 2026-01-08

**Authors:** Sadaki Shirao, Takaaki Arigami, Daisuke Matsushita, Masataka Shimonosono, Keishi Okubo, Masahiro Noda, Ken Sasaki, Kenji Baba, Takashi Tasaki, Akihide Tanimoto, Takao Ohtsuka

**Affiliations:** 1 Department of Digestive Surgery, Kagoshima University Graduate School of Medical and Dental Sciences, Kagoshima, Kagoshima, Japan; 2 Department of Pathology, Kagoshima University Graduate School of Medical and Dental Sciences, Kagoshima, Kagoshima, Japan

**Keywords:** gastric cancer, claudin 18.2, zolbetuximab, conversion surgery

## Abstract

**INTRODUCTION:**

Zolbetuximab, a monoclonal antibody targeting claudin (CLDN) 18.2, has been approved as a first-line treatment for patients with human epidermal growth factor receptor 2 (HER2)-negative and CLDN-positive advanced gastric cancer. Conversion surgery following chemotherapy has emerged as a promising strategy for improving outcomes in patients with stage IV gastric cancer. Herein, we report a case of CLDN-positive gastric cancer with peritoneal dissemination and cervical lymph node metastasis that was successfully treated with subtotal gastrectomy (sTG) following zolbetuximab-based chemotherapy.

**CASE PRESENTATION:**

A 73-year-old male was diagnosed with HER2-negative, CLDN-positive gastric cancer, accompanied by peritoneal dissemination and cervical lymph node metastasis. He received 6 cycles of zolbetuximab in combination with capecitabine and oxaliplatin as first-line chemotherapy. Primary tumor, peritoneal lesions, and lymph node metastases exhibited marked regression, including complete resolution of the metastatic cervical lymph nodes. Complete response was achieved according to the Response Evaluation Criteria for Solid Tumors. After confirming the disappearance of the peritoneal dissemination, the patient underwent sTG with D2 lymphadenectomy. Histopathological examination revealed a grade 2 therapeutic response and no residual lymph node metastasis. The postoperative course was uneventful, and the patient remained recurrence-free for 5 months after the conversion surgery.

**CONCLUSIONS:**

Zolbetuximab-based chemotherapy followed by conversion surgery is a promising therapeutic strategy for patients with CLDN-positive advanced gastric cancer.

## Abbreviations

CA
: carbohydrate antigen
CapeOX
: capecitabine and oxaliplatin
CEA
: carcinoembryonic antigen
CLDN
: claudin
CR
: complete response
CS
: conversion surgery
EGD
: esophagogastroduodenoscopy
HER
: human epidermal growth factor receptor
MST
: median survival time
RECIST
: response evaluation criteria for solid tumors
sTG
: subtotal gastrectomy
TJs
: tight junctions

## INTRODUCTION

Globally, gastric cancer ranks as the 5th most prevalent cancer and the fourth leading cause of cancer-related mortality.^1)^ According to the Japanese gastric cancer treatment guidelines, patients with unresectable, recurrent, or advanced gastric cancer are primarily treated with systemic chemotherapy.^2)^ While therapeutic advances have improved survival, stage IV disease—particularly when peritoneal seeding or distant lymph node metastasis is present—continues to carry a poor prognosis. These findings highlight the urgent need to develop effective therapeutic strategies. Zolbetuximab, a monoclonal antibody that targets CLDN18.2, was recently given approval as a 1st-line treatment for HER2-negative, CLDN18.2-positive advanced gastric cancer, and represents a new direction in molecular-targeted therapy for this disease.^3)^

CS following chemotherapy-induced tumor reduction has proven to be an effective approach, allowing for extended survival in certain patients initially diagnosed with unresectable advanced gastric cancer.^4–6)^ Combining systemic chemotherapy, molecular targeted therapy, and CS holds significant promise for enhancing outcomes in patients with advanced gastric cancer. Although the clinical efficacy of zolbetuximab-containing chemotherapy is still being established, to our knowledge, this is the first known case in which distant metastases resolved after zolbetuximab-based chemotherapy, thereby enabling successful CS.

## CASE PRESENTATION

**Fig. 1:**
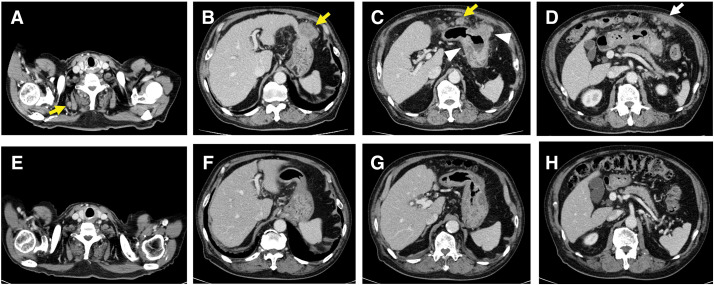
Enhanced CT images. (**A**–**D**) Before chemotherapy: A primary tumor penetrating the serosa in the lower gastric body (white arrowhead), swollen lymph nodes (yellow arrows), and peritoneal dissemination (white arrow) are observed. (**E**–**H**) After six cycles of CapeOX plus zolbetuximab: Tumor shrinkage, disappearance of lymph nodes, and resolution of peritoneal dissemination are evident. CapeOX, capecitabine and oxaliplatin

**Fig. 2:**
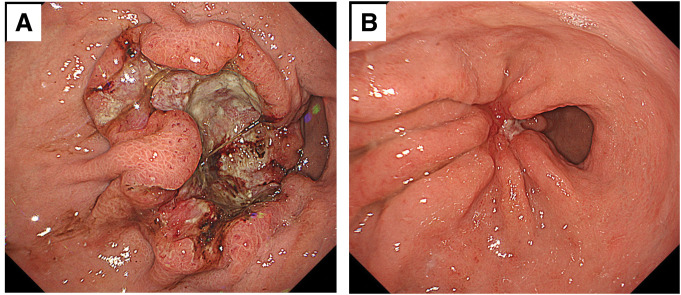
Esophagogastroduodenoscopy images. (**A**) Before chemotherapy: A type 2 tumor is seen on the greater curvature of the middle gastric body. (**B**) After six cycles of CapeOX plus zolbetuximab: The tumor has significantly regressed, and the ulcer base has contracted and become scarred. CapeOX, capecitabine and oxaliplatin

**Fig. 3:**
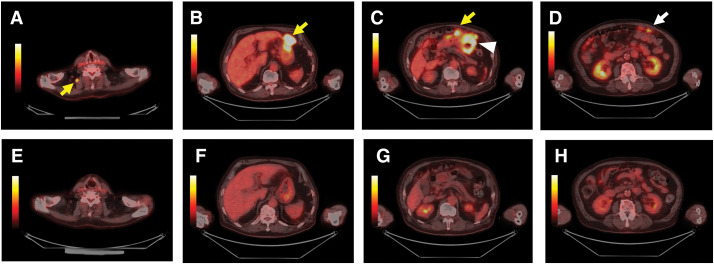
PET-CT images. (**A**–**D**) Before chemotherapy: Abnormal uptake is observed in the primary tumor (arrowhead), enlarged lymph nodes (yellow arrows), and the peritoneum (white arrow). (**E**–**H**) After six cycles of CapeOX plus zolbetuximab: Abnormal uptake in the primary tumor, lymph nodes, and peritoneum has resolved. CapeOX, capecitabine and oxaliplatin

A 73-year-old male was admitted to a local hospital due to abdominal pain and body weight loss. Contrast-enhanced CT showed a tumor in the lower gastric body that had penetrated the serosa. The CT scan also revealed peritoneal dissemination and multiple lymph node metastases, including one in the right cervical node (**Fig. 1A**–**1D**). EGD identified a type 2 tumor in the greater curvature of the middle gastric body (**Fig. 2A**). Histopathological examination of biopsied specimens, along with immunohistochemistry, showed well-differentiated adenocarcinoma with a HER2 score of 1+ and CLDN18.2 positivity. The patient was referred to our hospital where PET-CT showed abnormal uptake in the primary tumor and enlarged lymph nodes, including the right cervical node and peritoneum (**Fig. 3A**–**3D**). Based on these findings, the patient was clinically diagnosed with stage IV HER2-negative, CLDN-positive gastric cancer (cT4aN2M1).

The patient received zolbetuximab in combination with CapeOX as first-line chemotherapy. The regimen comprised a 3-week cycle of oral capecitabine (3000 mg/day) on days 1–14, intravenous oxaliplatin (130 mg/m^2^) on day 1, and zolbetuximab (800 mg/m^2^ for the first cycle, followed by 600 mg/m^2^) on day 1. After 6 cycles of CapeOX plus zolbetuximab, EGD revealed significant tumor shrinkage (**Fig. 2B**). CT and PET-CT demonstrated regression of the enlarged abdominal lymph nodes, disappearance of the right cervical lymph node metastasis, and resolution of peritoneal dissemination (**Figs. 1E**–**1H** and **3E**–**3H**). The target lesion shrinkage rate was 100%, indicating a CR according to the RECIST. Before chemotherapy, the patient’s serum tumor marker levels were 0.8 ng/mL for CEA and 91.4 U/mL for CA 19-9. After chemotherapy, the CEA level was 2.0 ng/mL and the CA19-9 level decreased to 9.2 U/mL.

**Fig. 4:**
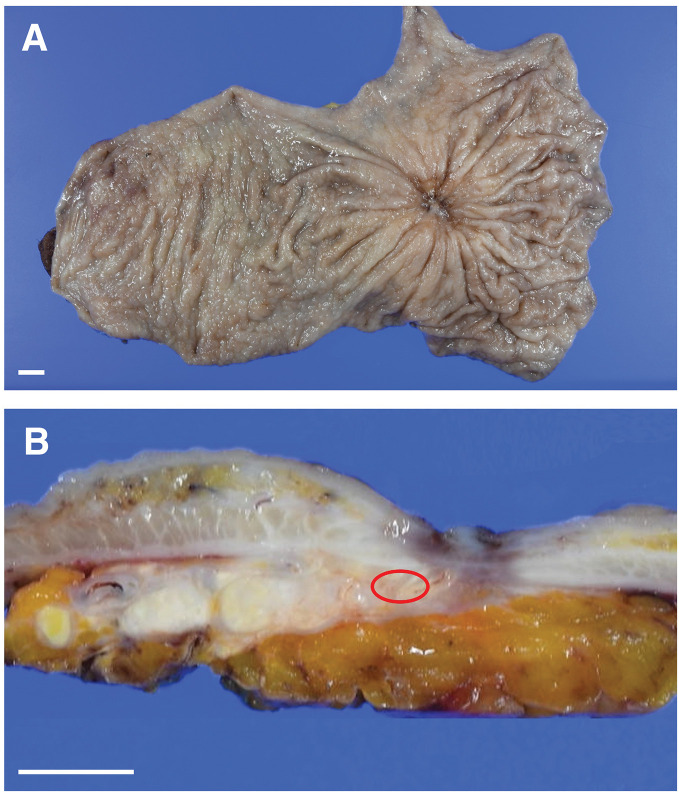
Macroscopic findings. (**A**) The formalin-fixed resected stomach showed a 15 × 15 mm ulcer with radiating mucosal folds. (**B**) On the cut surface of the stomach, the ulcer extended to the subserosal layer. The area of residual carcinoma is indicated by a red circle. Scale bars represent 1 cm.

**Fig. 5:**
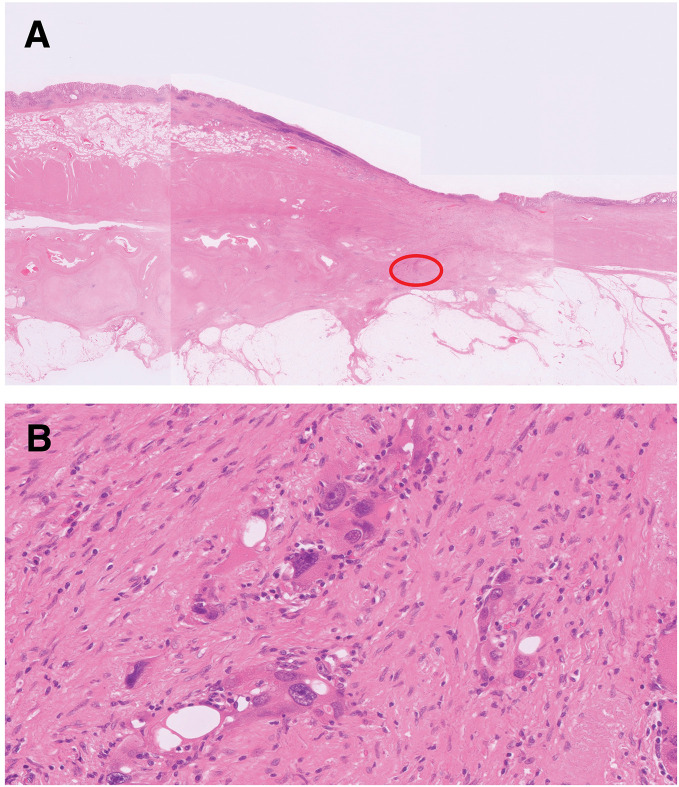
Microscopic findings. (**A**) Histopathological examination revealed marked fibrosis and a few carcinoma cells (red circle). (**B**) The residual carcinoma cells exhibited bizarre nuclei and vacuolar degeneration of the cytoplasm.

Following significant tumor regression, CS was considered to be one of the possible treatment options. Before the operation, the patient's serum total protein and albumin were measured at 6.5 g/dL and 3.6 g/dL, respectively. No visible peritoneal dissemination was observed, and ascitic cytology was negative. A laparoscopic sTG with D2 lymphadenectomy and Roux-en-Y gastrojejunostomy reconstruction, preserving a small remnant stomach, was performed because the tumor was located approximately 7 cm from the esophagogastric junction. Resection line was determined to ensure a proximal margin of 3 cm from the tumor scar, guided by intraoperative EGD. Intraoperative histopathological evaluation confirmed the absence of tumor invasion at the resection margins. The operation lasted for 471 min, with a total blood loss of 50 mL. Macroscopically, a 15 × 15 mm ulceration with radiating mucosal folds was observed in the resected stomach (**Fig. 4A** and **4B**). Histopathologically, a small number of residual carcinoma cells were identified within areas of marked fibrosis (**Fig. 5A** and **5B**). No carcinoma cells were detected in the dissected lymph nodes. These findings indicate a grade 2 histopathological response based on the 3rd English edition of the Japanese classification of gastric carcinoma.^7)^ The patient’s recovery after the operation was without complications, and they were discharged on POD 20. He was a current smoker, which delayed respiratory recovery and prolonged hospitalization. Therefore, adjuvant chemotherapy with oral S-1 was initiated on POD 45. No evidence of recurrence was observed at the 5-month postoperative follow-up.

## DISCUSSION

Claudins are a family of at least 27 transmembrane proteins that were first described by Tsukita and Furuse,^8)^ and are essential components of TJs. Among these, CLDN18.2 is a highly specific TJ protein expressed in the gastric mucosa.^9)^ Zolbetuximab is a genetically engineered chimeric monoclonal antibody that targets CLDN18.2 and induces tumor cell death via antibody- and complement-dependent cellular cytotoxicity.^10)^ Previous clinical trials, including the GLOW and SPOTLIGHT studies, have shown that the combination of zolbetuximab and chemotherapy led to a significant improvement in progression-free and overall survival in patients with CLDN18.2-positive, HER2-negative gastric adenocarcinoma.^11,12)^ In response to these findings, the 7th edition of the Japanese gastric cancer treatment guidelines, revised in March 2025, now recommends zolbetuximab-based chemotherapy as the first-line treatment for HER2-negative CLDN-positive gastric cancer. This report presents a case of stage IV HER2-negative, CLDN-positive gastric cancer with peritoneal dissemination and cervical lymph node metastasis in which sTG with a small remnant stomach was successfully treated with zolbetuximab-based chemotherapy. To the best of our knowledge, this is the first reported case of CS following zolbetuximab treatment in a patient with stage IV gastric cancer.

Although the GLOW and SPOTLIGHT studies provided limited data on whether the response rate induced by zolbetuximab was significantly superior to that of a placebo,^11,12)^ the present patient achieved CR to zolbetuximab-based chemotherapy, with a 100% reduction in multiple lymph node metastases and complete disappearance of peritoneal dissemination. Histopathological examination revealed a grade 2 tumor response in the primary lesion and no residual lymph node metastasis. According to the European Medicines Agency, the best percentage change from baseline in the sum of the diameters of target lesions was −47.9% in the zolbetuximab group and −38.4% in the placebo group based on combined analyses of the GLOW and SPOTLIGHT studies.^13)^ These findings suggest a promising antitumor effect of zolbetuximab as a molecularly targeted agent in patients with HER2-negative, CLDN-positive gastric cancer.

There is limited evidence on the effectiveness of CS in cases of unresectable gastric cancer responsive to chemotherapy.^14)^ In this case, we confirmed a CR radiologically according to RECIST criteria. In addition, intraoperative findings demonstrated macroscopic disappearance of peritoneal dissemination (P0) and negative ascitic cytology (CY0). Based on these findings, we determined that curative resection with negative resection margins (R0) could be achieved through CS in this patient. Previous case reports have shown that CS can be achieved in patients without CLDN18.2 expression following chemotherapy, including regimens with immune checkpoint inhibitors such as nivolumab and pembrolizumab.^15,16)^ Furthermore, a multicenter population-based cohort study reported that CS was performed in 8.8% of stage IV gastric cancer patients, regardless of CLDN18.2 expression, and was associated with improved survival, with R0 resection providing the best outcomes.^17)^ Therefore, further studies are warranted to compare the feasibility and outcomes of CS between patients with CLDN18.2-positive and -negative expression.

Currently, there is no consensus on whether surgery should be performed immediately upon confirming resectability or deferred until after additional chemotherapy. In this case, we opted for surgery after 6 cycles of chemotherapy. This decision was based on achieving a CR, which we interpreted as the point of maximum tumor regression. Furthermore, prolonged chemotherapy carries the risk of cumulative toxicities and the emergence of drug-resistant clones, potentially leading to tumor regrowth and loss of the surgical window. Accordingly, we prioritized minimizing the risk of missing the opportunity for curative resection over administering further consolidation cycles. Moreover, pathological examination revealed a histological response of grade 2 and the patient was pathologically diagnosed with stage IIA gastric cancer (T3N0M0). According to the Japanese gastric cancer treatment guidelines, 1 year of postoperative adjuvant chemotherapy with S-1 is recommended for pathological stage II gastric cancer.^2)^ Therefore, we planned to administer S-1 for 1 year. While the guidelines indicate that adjuvant chemotherapy is weakly recommended for stage IV gastric cancer following R0 resection, there is currently no clinical evidence regarding the optimal drug regimen or treatment duration in such cases. Consequently, further prospective studies are needed to clarify these issues.

There is a lack of research concerning the surgical management and long-term prognosis of stage IV gastric cancer patients who undergo CS after zolbetuximab-based chemotherapy. Notably, previous reports have described severe gastritis and hypoalbuminemia as adverse effects of zolbetuximab in patients with HER2-negative, CLDN-positive advanced gastric cancer.^18,19)^ In contrast, the present patient experienced an uneventful clinical course with no evidence of severe hypoalbuminemia or postoperative complications. However, for patients undergoing CS after zolbetuximab-based chemotherapy, it is essential to manage these possible adverse effects with care. A retrospective international cohort study (CONVO-GC-1) indicated that patients with stage IV gastric cancer who had surgery after chemotherapy had an MST of 36.7 months.^6)^ The MST for those who had curative R0 resection was 56.6 months.^6)^ In our patient's case, a complete R0 resection was performed with no residual tumor after CS, which points to a positive prognosis. Although the patient has remained recurrence-free for 5 months, individuals with a history of peritoneal dissemination are at high risk of recurrence, and a 5-month follow-up is insufficient to evaluate long-term survival. Accordingly, future large-scale cohort studies with extended follow-up periods are warranted to fully assess the prognostic effect of CS following zolbetuximab-based chemotherapy. Additionally, a randomized controlled phase III trial (JCOG2301) is currently underway to evaluate the clinical benefit of CS after palliative chemotherapy compared with palliative chemotherapy alone in patients with initially unresectable stage IV advanced gastric cancer who demonstrate a remarkable response to chemotherapy.^20)^ The results of this trial are eagerly awaited and are expected to provide critical insights into the role of CS in this patient population.

## CONCLUSIONS

We present a case of stage IV HER2-negative, CLDN18.2-positive gastric cancer with peritoneal dissemination and cervical lymph node metastasis. In this case, CS was successfully performed following zolbetuximab-based chemotherapy, which demonstrates a notable histopathological response. These findings suggest that CS following zolbetuximab-based chemotherapy may be a promising strategy for improving the prognosis of selected patients with stage IV HER2-negative, CLDN18.2-positive gastric cancer.

## DECLARATIONS

### Availability of data and materials

The datasets generated in this study are available from the corresponding author upon request.
